# Quantitative image analysis of immunohistochemical stains using a CMYK color model

**DOI:** 10.1186/1746-1596-2-8

**Published:** 2007-02-27

**Authors:** Nhu-An Pham, Andrew Morrison, Joerg Schwock, Sarit Aviel-Ronen, Vladimir Iakovlev, Ming-Sound Tsao, James Ho, David W Hedley

**Affiliations:** 1 Division of Applied Molecular Oncology, Ontario Cancer Institute/Princess Margaret Hospital, University Health Network, Toronto, Canada; 2 Department of Medical Biophysics, University of Toronto, Toronto, Canada; 3 Department of Laboratory Medicine and Pathobiology, University of Toronto, Toronto, Canada

## Abstract

**Background:**

Computer image analysis techniques have decreased effects of observer biases, and increased the sensitivity and the throughput of immunohistochemistry (IHC) as a tissue-based procedure for the evaluation of diseases.

**Methods:**

We adapted a Cyan/Magenta/Yellow/Key (CMYK) model for automated computer image analysis to quantify IHC stains in hematoxylin counterstained histological sections.

**Results:**

The spectral characteristics of the chromogens AEC, DAB and NovaRed as well as the counterstain hematoxylin were first determined using CMYK, Red/Green/Blue (RGB), normalized RGB and Hue/Saturation/Lightness (HSL) color models. The contrast of chromogen intensities on a 0–255 scale (24-bit image file) as well as compared to the hematoxylin counterstain was greatest using the Yellow channel of a CMYK color model, suggesting an improved sensitivity for IHC evaluation compared to other color models. An increase in activated STAT3 levels due to growth factor stimulation, quantified using the Yellow channel image analysis was associated with an increase detected by Western blotting. Two clinical image data sets were used to compare the Yellow channel automated method with observer-dependent methods. First, a quantification of DAB-labeled carbonic anhydrase IX hypoxia marker in 414 sections obtained from 138 biopsies of cervical carcinoma showed strong association between Yellow channel and positive color selection results. Second, a linear relationship was also demonstrated between Yellow intensity and visual scoring for NovaRed-labeled epidermal growth factor receptor in 256 non-small cell lung cancer biopsies.

**Conclusion:**

The Yellow channel image analysis method based on a CMYK color model is independent of observer biases for threshold and positive color selection, applicable to different chromogens, tolerant of hematoxylin, sensitive to small changes in IHC intensity and is applicable to simple automation procedures. These characteristics are advantageous for both basic as well as clinical research in an unbiased, reproducible and high throughput evaluation of IHC intensity.

## Background

Immunohistochemistry (IHC) for the evaluation of antigen expression as well as higher resolution methodologies for cytogenetic analysis are standard procedures for the diagnosis and prognosis of cancer and other diseases [1]. In recent years, cancer treatment response and disease progression increasingly rely on IHC to monitor changes in targeted antigens. Abundances of antigens have so far relied primarily on visual scoring and to a lesser extent computer-assisted image processing techniques [2]. A major advantage of computer techniques is the avoidance of inter-observer variability in interpreting subtle antigen level changes [3]. In addition, pattern recognition, which has been applied to image analysis of fluorescence *in situ *hybridization [4], may also be applied to IHC image analysis of compartmentalized antigen distributions, particularly with recent developments in self-learning computer programs [5]. Most computer-based techniques for IHC image analysis have so far had limited applicability due to several drawbacks including a need for specific software systems, often with considerable need for user input [6-9]. These image analyses have commonly been performed on the single assessment of 3,3'-diaminobenzidine (DAB) labeling for a variety of cytoplasmic markers. Consequently, misclassifications were frequently encountered when two or more chromogens with overlapping absorption spectra were used simultaneously [6]. Specialized color deconvolution algorithms can be applied to discriminate multiple spectra [10]. Alternatively, the recent application of spectral imaging offers an optimal method to capture and analyze images at multiple wavelengths [11]. However, digitally captured IHC brightfield images are usually stored as composites of three 8-bit monochromatic red, green and blue channels (RGB), which can be converted to the Cyan/Magenta/Yellow/Key (CMYK) or Hue/Saturation/Lightness (HSL) color space. Previously, components of the RGB [12], their normalization (nR, nG, nB; nRGB) [6], or HSL [5,8,13] have been applied towards IHC quantification.

In this study, we adapted a CMYK color model as a simple method to quantify IHC staining with three commonly used chromogens including 3-amino-9-ethylcarbazole (AEC), DAB, and NovaRed with hematoxylin counterstain. For simplicity, images of color bars representing IHC staining were used to characterize the performance of individual channels in the color models CMYK, RGB, nRGB and HSL. These results indicated advantages with the Yellow channel analysis, based on a CMYK color model. Therefore, the Yellow channel method was further tested on three applications. First, sensitivities of image analysis methods were compared in the IHC staining of phosphorylated signal transduction and transcription factor-3 (p-STAT3) in tumor xenografts. Following, the performance of a high throughput automated Yellow channel method was compared to observer-dependent methods, including positive color selection for a hypoxia marker carbonic anhydrase-IX (CA-IX)-labeled DAB and visual scoring for epidermal growth factor receptor (EGFR)-labeled NovaRed in the second and third applications, respectively.

## Methods

### Image acquisition and processing

Slides were scanned with a ScanScope CS (Aperio Technologies, Vista, CA) using brightfield imaging at 20× magnification. Specimen areas were selected and individual images were saved in a 24-bit RGB TIFF file format with a resolution of 1 μm/pixel using the ImageScope software (Aperio Technologies). The automated analysis of the TIFF image files was performed using the programming language IDL 6.3 (ITT Visual Information Solutions, Boulder, Colorado) [14,15] for IHC quantification using RGB [12], normalized RGB [6], HSL [8,13], and CMYK as described below.

### CMYK image analysis method

We adapted a CMYK model with maximum grey component replacement, a criterion in which the lowest brightness level is subtracted from all channels. This criterion effectively discriminates color differences by subtracting out the grey level, a level of equal intensity among the three channels (C, M, Y). Consequently, this subtraction sets one of the color channels to zero in each image. The selection of this display criterion for an image using a popular software Adobe^® ^Photoshop^®^7.0, involves the following sequence of menu selections: Edit, Color settings, Custom CMYK and Black generation set at Maximum. In this study, CMYK values were derived from the CMYK_Convert library procedure in IDL 6.3 using the equations:

(* indicates multiplication)

K (black) = 255 - maximum(R, G, B)

C = 255* [1 - R/(255 - K)] (if K = 255 then C = 0)

M = 255* [1 - G/(255 - K)] (if K = 255 then M = 0)

Y = 255* [1 - B/(255 - K)] (if K = 255 then Y = 0)

Mean intensity measurements in regions of interest were computed without the use of positive color intensity thresholds. However, proportions of labeled area in tissue were determined with a positive range intensity of 21+ and 93+ for the Yellow and nRed channels, respectively.

### Xenograft models

Spectral characteristics of AEC, DAB and NovaRed were displayed with the different color models using color bars that were generated from a range of representative IHC staining derived from the xenograft studies described below, as well as additional IHC archived slides that showed a greater range of staining intensity.

Changes in STAT3 phosphorylation (p-STAT3) levels were associated with different conditions, including growth factor stimulation and sampling techniques as previously described [16]. Formalin-fixed paraffin embedded specimens were obtained to compare IHC staining using different chromogens AEC, DAB or NovaRed, and hematoxylin counterstain. Briefly, a tumor xenograft was excised and divided into two equal parts. One part was further divided and subjected to immediate formalin fixation, or protein extraction for Western blot analysis [17]. The second part was incubated with 50 ng/ml epidermal growth factor (EGF, Sigma-Aldrich, Oakville, ON) in PBS for 20 minutes prior to specimen analysis. The antibody ^S727^p-STAT3 (BD Biosciences, Mississauga, ON) was used for Western blotting at a dilution of 1:100, and anti-GAPDH used at a dilution of 1:5000 (Ambion, Foster City, CA). Protein bands were detected with ECL plus fluorescence, imaged with a Typhoon system and their intensities were quantified (mean intensity × band area) and normalized with GAPDH using ImageQuant5.2 software (GE Healthcare, Little Chalfont, Buckinghamshire, UK).

Second, p-STAT3 levels were also quantified in formalin-fixed paraffin embedded sections of fine-needle samples obtained from a tumor xenograft. These samples were subjected to either immediate or delayed fixation after 5, 20 or 60 minutes, which caused an increase in p-STAT3.

### Clinical test sets for comparison with CMYK model

Image analysis was performed on two clinical image data sets collected for other histopathological studies. The first study comprised of a total of 414 sections obtained from three adjacent sections of 138 frozen uterine cervical carcinoma biopsies. Carbonic anhydrase-IX-labeled DAB was previously evaluated with a positive color selection method [18]. In brief, a visual selection of a range of positive brown color pixels was performed using Adobe^®^Photoshop^® ^7.0 and batched analyzed using the IDL 6.3 programming language. Image analysis was performed in areas designated by tumor masks.

The second study comprised of a total of 282 formalin-fixed and paraffin embedded NSCLC biopsies arranged in 8 tissue microarrays which were IHC-stained for EGFR-labeled NovaRed [19]. Three cores of 0.6 mm diameter represented each case with evaluation completed on cases represented by at least 2 cores (*n *= 256). Two evaluators visually scored with a scale 0–3 (0 for absence of membrane staining in cancer cells) and their scores were strongly associated (r = 0.97). Thus, the mean of core scores was used as the final score for individual cases. Image analysis was performed in areas designated by whole tissue core masks since anti-EGFR demonstrated high specificity for cancer cells.

### Immunohistochemistry

Specimens were processed with routine protocols using anti-^S727^p-STAT3 (BD Biosciences), anti-CA-IX (a generous gift of Dr. Adrian Harris, University of Oxford, UK) [15] and anti-EGFR (clone 31G7, Zymed Technologies, Invitrogen, Burlington, ON). The Idetect Ultra HRP Detection System (ID Labs Inc, London, Ontario) was used to visualize either DAB (Dako Corp, Carpinteria, CA), NovaRED or AEC (Vector Laboratories). Counterstaining was performed with Gill modified hematoxylin (Harleco^®^).

## Results

### IHC spectral characteristics

**Figure 1:**
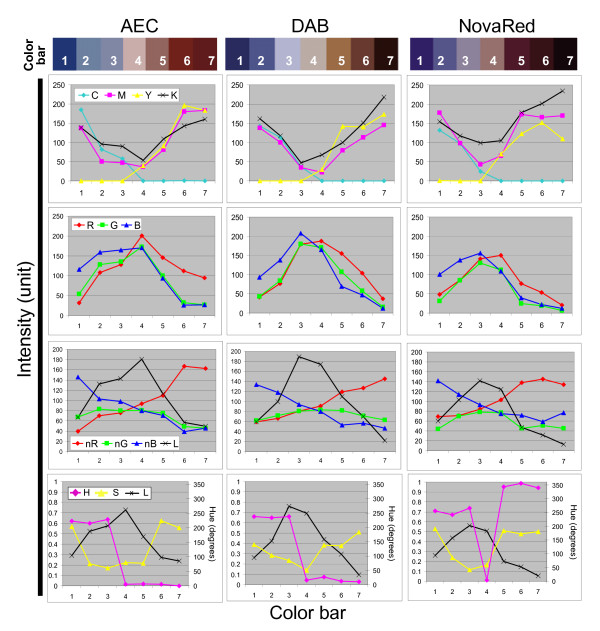
**IHC spectral characteristics**. The color bars designated 1–7 display representative IHC staining, from least to most, using AEC, DAB, or NovaRed with hematoxylin counterstained nuclei. Graphs on sequential rows depict the IHC colors defined by the individual channel intensity of CMYK, RGB, nRGB and HSL color model.

Four color models, CMYK, RGB, nRGB and HSL, were applied to examine the spectral characteristics of AEC, DAB, NovaRed and hematoxylin counterstain (Figure 1). Chromogen staining in hematoxylin counterstained specimen ranged from absent (color bars 1–3, Figure 1) to present (color bars 4 to 7, Figure 1) for the respective chromogens in the nuclear compartment sampled. Increasing chromogen intensity was similar to intensities obtained in the absence of hematoxylin counterstain [See additional file 1]. However, in the presence of hematoxylin counterstain (Figure 1) the intensity levels of most channels overlapped between hematoxylin and chromogen colors except for those characterized by the Yellow channel, normalized Red channel (nR) or normalized Blue channel (nB). Interestingly, highest chromogen intensity levels saturated on the Yellow, nR or nB scale in either the presence or absence of hematoxylin (Figure 1 and Additional file 1). This observation suggests that it is important to remain below chromogen saturation levels for a direct correlation with intensity levels.

Unique features of the presented Yellow channel included an automatic setting at zero for the hematoxylin counterstain as well as a greater utilization of the 0–255 intensity scale compared to nR or nB. These characteristics demonstrated that the Yellow channel achieved a high contrast between different chromogen intensities as well as between the tested chromogens and the hematoxylin counterstain. The performance of the CMYK Yellow channel method was further tested in three applications which used other automated and observer-dependent methods.

### STAT3 activation

**Figure 2:**
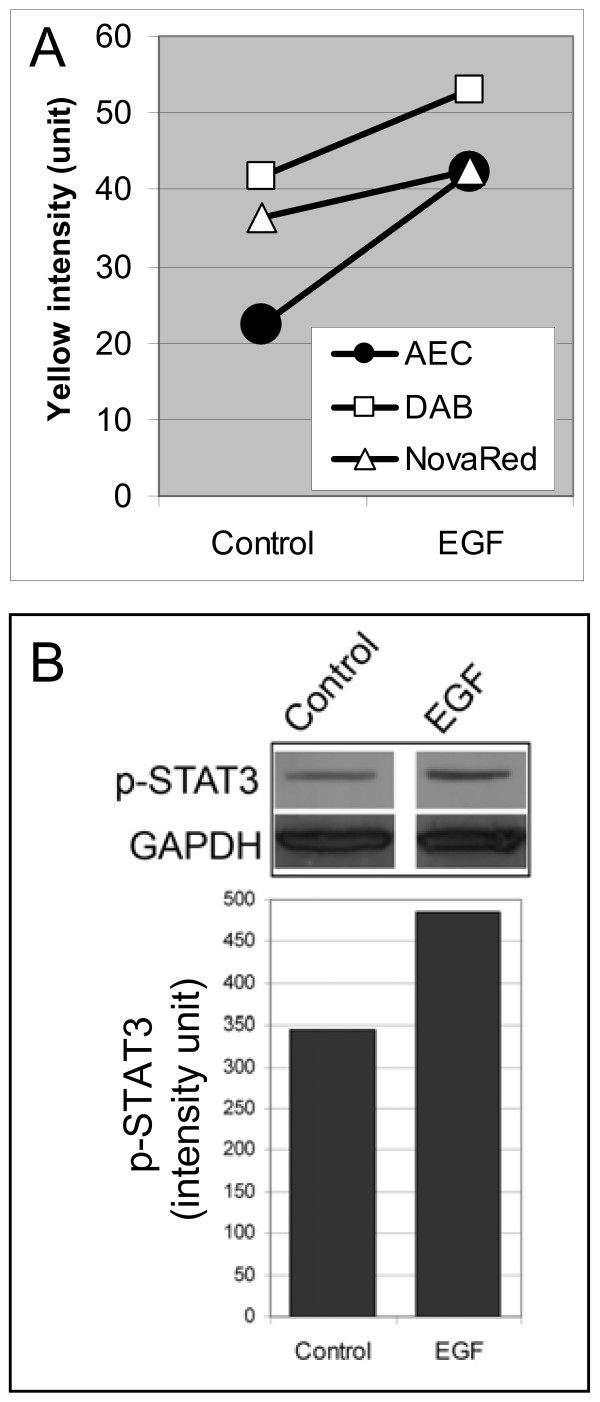
**STAT3 activation in EGF-stimulated tumor tissue**. (A) Yellow channel image analysis was performed on consecutive sections detecting p-STAT3 labeled with each of the three chromogens and counterstained with hematoxylin. (B) An increase in p-STAT3 was detected by Western blotting.

Levels of activated STAT3 induced by EGF were quantified in IHC-stained xenografts using Yellow channel image analysis (Figure 2A). Despite considerable heterogeneity in activated STAT3 IHC staining as displayed from the images [see additional file 2], an increase in staining is noticeable after EGF stimulation within the viable tumor area which is in agreement with Western blotting (Figure 2B). A comparison of different color channel analyses showed that measurements obtained from the Yellow channel were consistently more sensitive to increases in staining intensity, particularly with the AEC substrate (Table 1).

**Table 1:** Activation of STAT3

Channel	EGF Stimulation (%)		
	**AEC**	**DAB**	**NovaRed**

**Yellow**	180	130	120
**nRed**	106	103	102
**Key**	110	120	110

Mean IHC-p-STAT3 intensity was quantified using different color channels. Effects of EGF stimulation were expressed relative to control.

**Figure 3:**
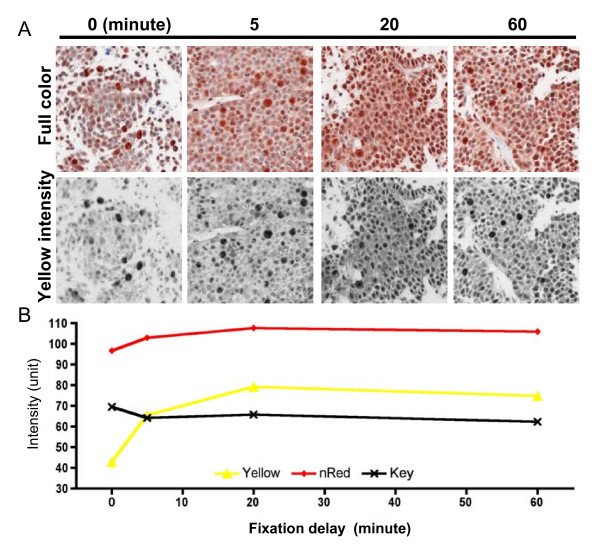
**STAT3 activation after delayed tissue fixation**. (A) Phosphorylated STAT3-labeled AEC and hematoxylin counterstained representative images are shown in full color and Yellow intensity as shown in grayscale. (B) The mean Yellow intensity within the entire area of the four specimens shows a staining increase with a maximum at 20 minutes. This increase was smaller in magnitude and undetectable in results obtained from the normalized Red and the Key channel, respectively.

Furthermore, image analysis was applied to a series of small fine-needle samples which displayed differences in STAT3 activation (Figure 3A). Mean Yellow intensity measurements showed an increase in staining intensities with a maximum at 20 minutes (Figure 3B). This result was in agreement with a previously observed biological trend [16]. Measurements of IHC staining showed greater magnitudes of change using Yellow compared to the normalized Red channel analysis. Additionally, the Yellow channel was relatively unaffected by the hematoxylin which resulted in a slightly higher Key channel intensity at the initial compared to the last time point.

### Positive color selection analysis compared to CMYK

**Figure 4:**
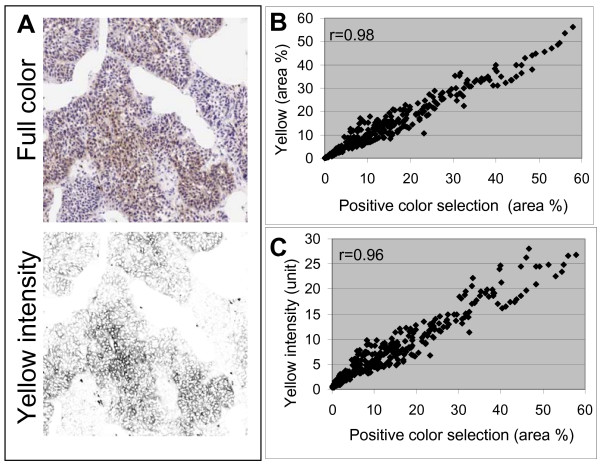
**Positive color selection compared to CMYK analysis**. (A) A representative field of 411 sections of cervical carcinoma shows CA-IX-DAB labeling with hematoxylin counterstain in full color and in Yellow intensity as shown in grayscale. (B) Percentages of IHC-labeled area as well as (C) mean intensity were analyzed using the Yellow channel and either results display a strong association with results obtained from positive color selection method.

The more arbitrary method using positive color selection of CA-IX levels [18] was compared to results obtained using Yellow channel analysis (Figure 4A). Since CA-IX staining was characterized by a heterogeneous pattern rather than a ubiquitously distribution in all cells, the fraction of labeled cells was of primary interest. Positively labeled area fractions were strongly correlated between the two methods (Figure 4B). Although the mean Yellow intensity measurements were also strongly correlated with area fractions using positive color selection analysis (Figure 4C), the slight decrease in the correlation of these parameters at the higher end suggests higher variability in CA-IX levels in tumors with larger fractions of CA-IX labeled areas. Strong associations were obtained between results from positive color selection and the Yellow or the normalized Red channel (Table 2). Measurements of percent of positive area showed strong associations among all methods as was expected, since a fixed positive threshold/range intensity was used in each case. However, measurements from the positive color selection method have a higher association with intensity measurements from the Yellow rather than the normalized Red channel. This result suggested that the Yellow intensity provides an observer bias-free measurement with relatively high accuracy.

**Table 2:** A comparison of image analysis methods

	**Yellow channel**		**nRed channel**	
	Intensity (mean)	% Positive area	Intensity (mean)	% Positive area

**Positive color selection **(% positive area)	0.96	0.98	0.84	0.98

Values (correlation coefficient, r) indicate strength of a linear relationship between results from positive color selection and Yellow or normalized Red analysis method, where a maximum positive is r = 1.

### Pathological visual scoring compared to CMYK

**Figure 5:**
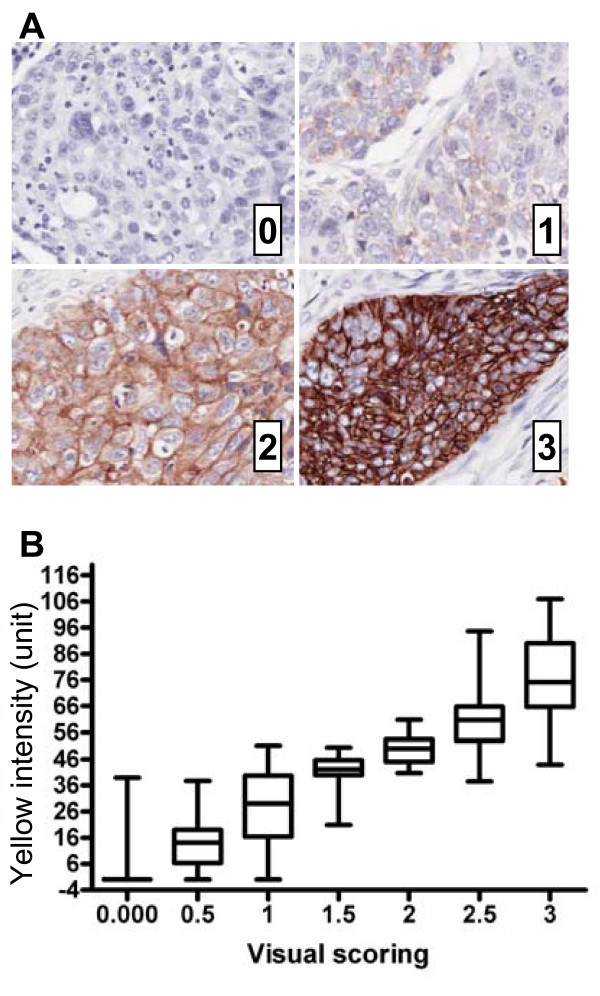
**Visual scoring compared to CMYK analysis**. (A) Representative images of 256 NSCLC biopsies show plasma membrane specific EGFR-NovaRed labeling with visual scores ranging from 0–3. (B) A box and whiskers graph (median, 25% to 75% box range, min and max whiskers) representation of mean Yellow intensity of specimens shows a direct relationship with the categorical visual scores.

The mean IHC intensity of each biopsy based on a visual scoring performed by two pathologists (Figure 5A) showed a direct relationship (r = 0.89) with mean Yellow intensity measurements (Figure 5B). Interestingly, a higher variability characterized the lower and the upper ends of the scoring range (e.g. 0–1 and 2.5–3) compared to the middle of the scoring categories. This result suggests some cases at either end of the scale were misclassified and might be due to inconsistencies in the visual scores, or inclusion of non-specific staining with an automatic analysis based on whole tissue core. However, the majority of the cases from the different scoring categories were grouped within the non-overlapping top and bottom 25^th ^percentile of the Yellow intensity values.

## Discussion

Levels of IHC stains are increasingly used to monitor biological disease markers during cancer treatment and disease progression. This image analysis study tested the use of the Yellow channel of a CMYK color model for IHC quantification. Although the programming language IDL 6.3 was used to automate the analysis process here, the availability of the CMYK model with maximum grey component replacement function in popular image processing software packages makes this method readily accessible.

Spectral characterization of the chromogens AEC, DAB and NovaRed in the absence of the hematoxylin counterstain showed a relationship with at least one channel of all color models, consistent with the RGB and HSL results in previous reports [6,8,12]. However, in the presence of the hematoxylin counterstain the Yellow channel analysis method applied here achieved the best contrast on a 0–255 scale, between chromogen and hematoxylin as well as for different chromogen intensities. These characteristics of the Yellow channel resulted in a higher sensitivity towards detecting subtle IHC intensity changes relevant to markers associated with treatment response, prognosis and pathobiology of cancers in the examples presented in this paper.

The least amount of observer-dependent input was applied to the image analysis process. Analyses were performed in regions of interest with the omission of obvious dark artifacts which may occur due to mixing with endogenous pigments, debris, tissue drying and other method-dependent variables as recognized in such tissue processing techniques [2,20]. Although a comprise on the intensity scale occurs when the IHC stain is near black as presented in the results, the saturated chromogen intensities remained within the higher levels of the intensity scale and therefore was likely negligible. Alternatively, independent of intensity levels, measurements were also evaluated based on proportions of labeled area, but it is dependent on observer selection of positive intensity ranges.

Antigen expression in tissue sections can be expressed as either the mean staining intensity or the stained area fraction. The mean staining intensity measurements described in this paper show changes in relative antigen expression when these were ubiquitously expressed, or equally distributed in compartment(s) of interest. These antigen characteristics suggest that other frequently IHC-labeled antigens such as proliferation and apoptosis markers can also be quantified using image analysis methods such as Yellow channel in addition to conventional estimates of their presence or absence. These markers have been informative for the further sub-classification of cancer stages identified using standard histology [21,22]. However, in scenarios where differences in cellular distributions are important for disease profiling [23] recent developments in pattern recognition programs are possible solutions [5].

The analysis of different IHC applications here demonstrated that the Yellow-CMYK channel method provides consistent results as well as higher performance for IHC quantification compared to other automated and manual techniques. The Yellow channel has several advantages, including its applicability to different chromogens, tolerance of hematoxylin, greater utilization of the intensity scale and readiness for automation. In particular, the mean Yellow intensity measurements are independent of arbitrary threshold selection. These advantages are important to IHC-based analysis in basic as well as clinical research [24] where the biological changes as well as methodological variances need to be quantified in an unbiased, reproducible and high throughput manner.

## Abbreviations

AEC, 3-amino-9-ethylcarbazole; CMYK, cyan/magenta/yellow/black; DAB, 3,3'-diaminobenzidine; HSL, hue/saturation/lightness; IHC, immunohistochemistry; RGB, red/green/blue

## Competing interests

The author(s) declare that they have no competing interests.

## Authors' contributions

NAP conceived and performed the study. AM incorporated the use of a maximum grey component replacement in the CMYK method and automated the analysis. JS participated in data acquisition and analysis of the xenograft experiments. SAV and VI contributed the clinical image data sets and participated in the analysis. MST participated in the coordination of the study. JH performed the immunohistochemistry. DWH participated in the design and helped to draft the manuscript. All authors read and approved the final manuscript.

## Supplementary Material

Additional File 1 Supplementary Figure 1 Chromogen spectral characteristics without hematoxylin counterstain. Color bars representing single IHC chromogens are defined by the individual color channels from the models CMYK, RGB, nRGB and HSL. Click here for file

Additional File 2 Supplementary Figure 2 Activated STAT3 in tumor xenograft. Representative images of IHC staining for p-STAT3. Click here for file
