# Supplementary material for: Quantitative image analysis of immunohistochemical stains using a CMYK color model
Source: Diagn Pathol. 2007 Feb 27;2:8. doi: 10.1186/1746-1596-2-8 (PMC1810239; doi:10.1186/1746-1596-2-8)
Supplement: Additional File 1 — Supplementary Figure 1 Chromogen spectral characteristics without hematoxylin counterstain. Color bars representing single IHC chromogens are defined by the individual color channels from the models CMYK, RGB, nRGB and HSL. [file 1746-1596-2-8-S1.pdf]

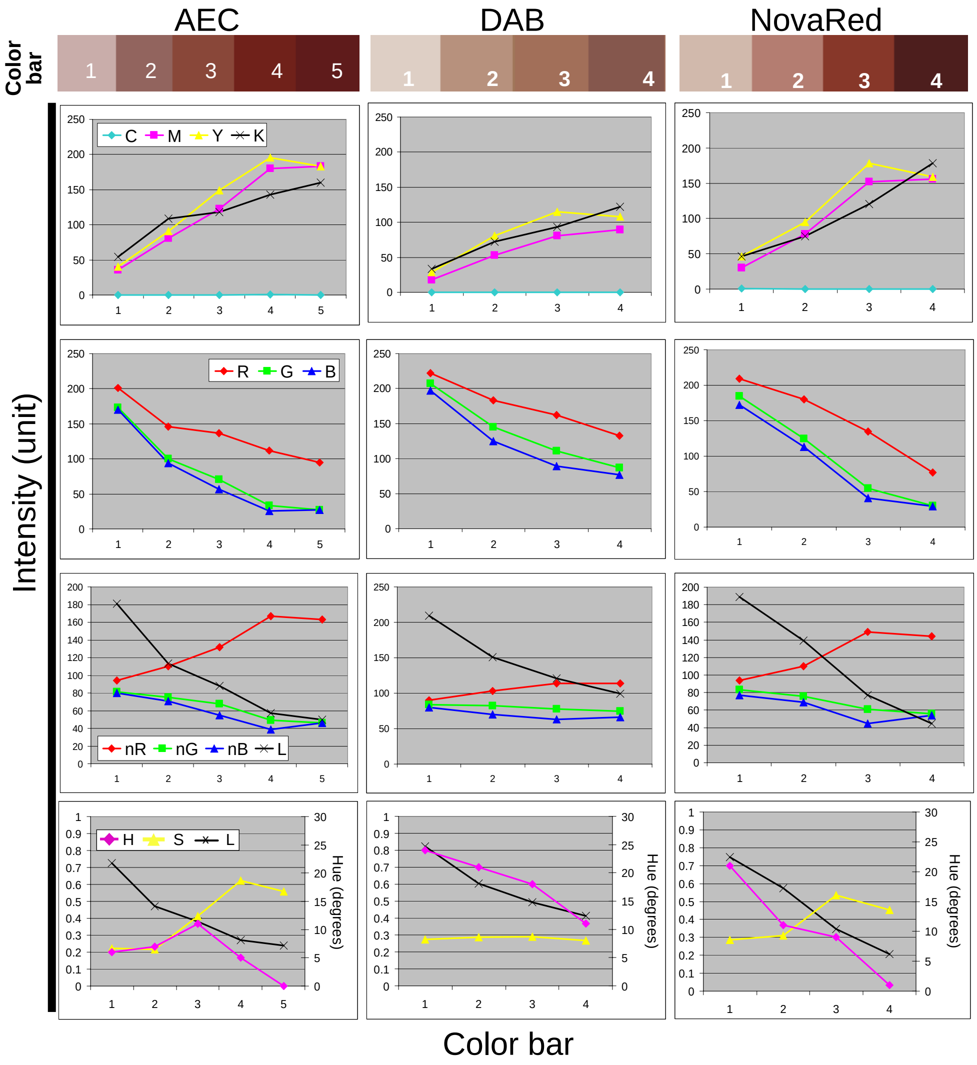

Supplementary Figure 1 Chromogen spectral characteristics. Color bars representing IHC stains without hematoxylin counterstain are defined by the individual channels of the different color models, CMYK, RGB, nRGB and HSL in each row.
