# Supplementary material for: Quantitative image analysis of immunohistochemical stains using a CMYK color model
Source: Diagn Pathol. 2007 Feb 27;2:8. doi: 10.1186/1746-1596-2-8 (PMC1810239; doi:10.1186/1746-1596-2-8)
Supplement: Additional File 2 — Supplementary Figure 2 Activated STAT3 in tumor xenograft. Representative images of IHC staining for p-STAT3. [file 1746-1596-2-8-S2.pdf]

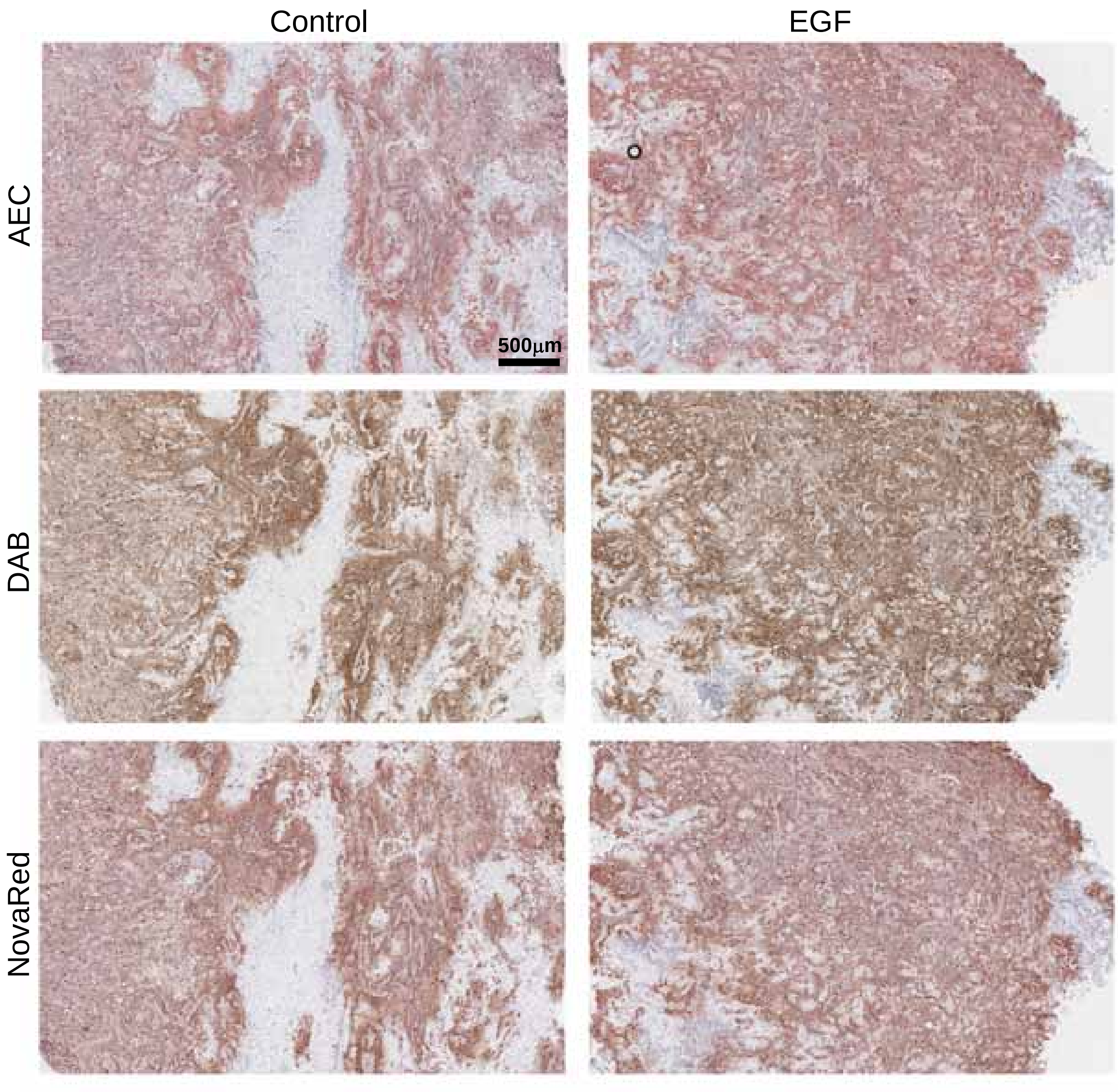

Supplementary Figure 2. Levels of ^{S727}p-STAT3 was examined in a tumor xenograft specimen divided for EGF stimulation compared to control. Serial sections were stained with the different chromogens and hematoxylin counterstained.
